# Supplementary material for: Inhibitory PD-1 axis maintains high-avidity stem-like CD8+ T cells
Source: Nature. 2025 Nov 26;649(8095):194–204. doi: 10.1038/s41586-025-09440-x (PMC12727512; doi:10.1038/s41586-025-09440-x)
Supplement: Supplementary file 1 — Detailed descriptions of image analysis methods [file 41586_2025_9440_MOESM1_ESM.pdf]

nature portfolio

https://doi.org/10.1038/s41586-025-09440-x

Supplementary information

Inhibitory PD-1 axis maintains high-avidity stem-like CD8$^+$ T cells

In the format provided by the authors and unedited

Supplementary Methods:

Image Analysis

Due to the very large number of cells acquired during 3D volumetric imaging (>1 million segmented cells), use of a conventional image processing pipeline conducted on dedicated workstations is impractical due to the exponential amount of data captured compared to thin tissue sections, with the length of time for processing increased by orders of magnitude (days-weeks).

To efficiently process such large 3D volumetric datasets, a Python-based computational pipeline was developed and optimized for distributed processing, in which the images were split into multiple sub-blocks to be processed in parallel on the NIH High Performing Computational (HPC) Biowulf cluster.

Image Pre-processing

Each raw Leica image file (.lif) was first converted into Imaris software format (.ims) using the Imaris File Converter tool (Bitplane). The ims format is a HDF5-based container and enables image processing steps to be seamlessly integrated into custom Python-based scripts.

The antibody staining panel was designed, together with the use of tunable excitation laser lines with tunable detection filters, to minimize spectral spillover and no further processing is required for most channels. For GFP/XCR1-venus signal crosstalk, pairwise compensation was performed as described previously[76]. Briefly, the spillover coefficient

was first determined by sampling a small area containing GFP+ (fluorophore A) and Venus+ (fluorophore B) cells, where the spillover coefficient is defined as:

$$S_{A>B} = \frac{A_{ch\_B}}{A_{ch\_A}}$$

where $S_{A>B}$ is the spillover coefficient for fluorophore A into channel B,

A_{ch_B} is the mean intensity of fluorophore A detected in channel B, and

A_{ch_A} is the mean intensity of fluorophore A detected in channel A as determined with a single-color control, or in cells with mutually exclusive fluorophores.

The reciprocal spillover coefficient for fluorophore B into channel A was also obtained:

$$S_{B>A} = \frac{B_{ch\_A}}{B_{ch\_B}}$$

Pairwise compensation can now be solved by with the following formula:

$$B_{comp} = \frac{D_{ch\_B} - (S_{A>B} \times D_{ch\_A})}{1 - (S_{A>B} \times S_{B>A})}$$

where B_{comp} is the compensated intensity for channel B,

D_{ch_B} is the intensity detected in channel B, and

D_{ch_A} is the intensity detected in channel A.

Compensation involving more than two colors were performed by multiplying the intensities of all overlapping channels with an inverse matrix of the spillover coefficients of all overlapping channels[76].

Intensity attenuation along z-axis of each channel was then examined by plotting the mean intensity of each slice (thresholded above background) along the z-axis. In most cases, intensity attenuation was minimal across the entire 3D volume and no further correction was required. In some images where a significant intensity drop was observed, a reference z-slice was used and the intensity of each z-slice was multiplied to match the mean intensity of the reference slice. Further trimming of the z-stack was performed in rare cases where high non-specific staining was observed near the surface of the tissues and when antibody staining did not fully penetrate the entire 3D volume.

Segmentation

Stardist3D was used for segmentation of lymphoid-shaped cells. A modified version was developed to enable accelerated processing of large volumetric datasets on the NIH HPC Biowulf cluster.

First, a training dataset was annotated semi-automatically. Briefly, a small volume of an image containing a representative distribution of ~100-200 cells was cropped and preliminary segmentation was performed using seeded watershed 3D segmentation. The cell labels were then manually corrected in *napari* and used as annotated labels for training a custom Stardist3D model. Nuclear Ki-67 staining was typically used as the segmentation channel, but for certain images, GFP+ fluorescent cells were segmented with the GFP channel.

A new custom model was trained for each new set of imaging data. The prediction steps were then performed on the NIH HPC Biowulf cluster with the full-sized segmentation

channel. Briefly, training and prediction of the 3D U-Net model was performed on GPU nodes, whereas the CPU-intensive non-maximal suppression and labeling steps were processed on multi-purpose CPU nodes. The output segmentation image containing individual cell labels (nuclear masks) was then used for downstream processing.

For CD45.1+ or CD45.2+ donor cells, segmentation was performed on the CD45.1/CD45.2 membrane marker channel using the pre-trained 'cyto3' model from Cellpose and with the '2D stitching' mode that iterates over the z-slices to construct 3D masks. The values used for the parameter 'diameter' is between 25 to 32.5 (depending on the xy pixel resolution), 'flow_threshold=0.6' and 'stitch_threshold=0.5'. These parameters adequately segmented the 3D volumes of cells based on CD45.1/2 membrane staining.

To accelerate Cellpose segmentation on large 3D volumetric images, the prediction and post-processing steps were performed on the NIH HPC Biowulf cluster. 3D images were split into multiple overlapping blocks of 512 x 512 x 48 pixels in x, y, z dimensions, plus an additional overlapping region of 64 x 64 x 6 pixels in each direction. Segmentation was first performed independently on each block with multiple GPU nodes in parallel, followed by the removal of redundant cell masks from the overlapping regions by iterating through each overlapping edge between the 3D segmented blocks. Briefly, overlapping cell masks between each block were compared and the block containing the largest cell mask volume will be retained, while the redundant cell masks in the other blocks were culled. These steps ensure that each cell only has one segmented mask allocated to a single block despite the overlapping regions between adjacent blocks.

Segmentation mask generation and data extraction

Segmentation labels, if trained on nuclear stains, will not contain the membrane and cytoplasmic regions of the cells. To enable staining intensity information to be extracted, a membrane/cytoplasmic cell mask was first generated through morphological filtering using the *scikit-image* library, with dilation (radius=6) and erosion (radius=3) of the initial nuclear mask, followed by subtraction of the eroded mask from the dilated mask to create a new membrane/cytoplasmic mask. For segmentation labels of membrane channel, morphological filtering steps with dilation (radius=1) followed by erosion (radius=8) were performed to create a similar membrane/cytoplasmic mask. Gaussian filtering was performed to apply a weighted gradient to the masks.

To obtain the voxel intensities of single cells, cell boundary coordinates of each segmented cell were used to extract a 3D patch of the image channels (a rectangular cuboid containing fluorescent markers of the single cell). This 3D patch was multiplied with the masks (membrane and/or nuclear) to retain only the voxels within the cell mask. The mean intensity of each fluorescent channel was obtained by dividing the summed voxel intensities by the sum of mask values. The final output is a data array containing the cell coordinates (x, y, z), mean marker intensities of each channel as output values. User-defined custom mathematical functions can also be added as a module to generate additional parameters if desired. Due to the large number of segmented cells, a computational pipeline optimized for distributed processing on HPC clusters was developed and deployed for routine operations.

Histocytometric gating, quantification of protein expression and subset classification

Raw data extracted based on cell masks and protein marker channels, together with their positional coordinates, were further analyzed using custom Python-based tools. Briefly, mean intensity of protein markers of single cells were projected on two-dimensional histocytometric plots visualized using *matplotlib* library, and gating was performed to select subsets of cells for further analyses. Activated donor OT-I cells were selected based on co-expression of Ki-67 and GFP/CD45.1. For polyclonal activated CD8+ T cells, the subset was gated based on Ki-67 and CD8b expression. To enhance the membrane staining signal from background noise, the ratio of mean intensity in the membrane mask (protein signal) versus the nuclear mask (background noise) was used to obtain a signal-to-noise ratio value. Alternatively, the mean nuclear intensity (background noise) of a membrane marker was subtracted from the mean membrane intensity (protein signal) to perform background subtraction. Negative values after the subtraction (indicating signal-to-noise ratio of <1) were set to zero.

Further subsets were generated based on TCF-1 and PD-1 expression to define PD-1$^+$ and PD-1$^-$ TCF-1$^+$ T_{SL}, as well as TCF-1$^-$ T_{EFF}. The spatial distribution of each subset was then visualized using Imaris 10.0 and XT extension (Bitplane) through creating new Spots layers of each subset. Each Spots layer corresponding to a subset was then set to a different color for visualization purpose.

References

76 Gerner, M. Y., Kastenmuller, W., Ifrim, I., Kabat, J. & Germain, R. N. Histo-cytometry: a method for highly multiplex quantitative tissue imaging analysis applied to dendritic cell subset microanatomy in lymph nodes. *Immunity* **37**, 364-376 (2012). https://doi.org/10.1016/j.immuni.2012.07.011
