# Supplementary material for: Inhibitory PD-1 axis maintains high-avidity stem-like CD8+ T cells
Source: Nature. 2025 Nov 26;649(8095):194–204. doi: 10.1038/s41586-025-09440-x (PMC12727512; doi:10.1038/s41586-025-09440-x)
Supplement: Supplementary file 2 — Reporting Summary [file 41586_2025_9440_MOESM2_ESM.pdf]

nature portfolio

Corresponding author(s): Ronald N. Germain, Jyh Liang Hor

Last updated by author(s): Jun 12, 2025

Reporting Summary

Nature Portfolio wishes to improve the reproducibility of the work that we publish. This form provides structure for consistency and transparency in reporting. For further information on Nature Portfolio policies, see our Editorial Policies and the Editorial Policy Checklist.

Statistics

For all statistical analyses, confirm that the following items are present in the figure legend, table legend, main text, or Methods section.

n/a	Confirmed	
☐	☒	The exact sample size (*n*) for each experimental group/condition, given as a discrete number and unit of measurement
☐	☒	A statement on whether measurements were taken from distinct samples or whether the same sample was measured repeatedly
☐	☒	The statistical test(s) used AND whether they are one- or two-sided<br>*Only common tests should be described solely by name; describe more complex techniques in the Methods section.*
☐	☒	A description of all covariates tested
☐	☒	A description of any assumptions or corrections, such as tests of normality and adjustment for multiple comparisons
☐	☒	A full description of the statistical parameters including central tendency (e.g. means) or other basic estimates (e.g. regression coefficient) AND variation (e.g. standard deviation) or associated estimates of uncertainty (e.g. confidence intervals)
☐	☒	For null hypothesis testing, the test statistic (e.g. *F*, *t*, *r*) with confidence intervals, effect sizes, degrees of freedom and *P* value noted<br>*Give P values as exact values whenever suitable.*
☒	☐	For Bayesian analysis, information on the choice of priors and Markov chain Monte Carlo settings
☒	☐	For hierarchical and complex designs, identification of the appropriate level for tests and full reporting of outcomes
☒	☐	Estimates of effect sizes (e.g. Cohen's *d*, Pearson's *r*), indicating how they were calculated

Our web collection on statistics for biologists contains articles on many of the points above.

Software and code

Policy information about availability of computer code

Data collection	Flow cytometry data were collected using Facs Diva v8.0.3 (BD Biosciences). Confocal microscopy data using Leica LAS X software (v3.5.7 for SP8, v4.6.0 and v4.8.0 for STELLARIS).
Data analysis	Imaging processing and analysis pipeline comprised the use of custom Python-based scripts (available on Github: https://github.com/jlhor/3d-imaging-pipeline), open source (Fiji ImageJ v1.54f) and commercial softwares (Imaris v10.0.0). Flow cytometry data were analyzed using FlowJo v10. Statistical tests were performed in Graphpad Prism v9. Linear modeling was performed using R v4.2.0. Graphs were plotted in Prism v9 and edited for appearance using Adobe Illustrator 28.5. Animations were generated using napari-animation package and edited with Davinci Resolve 18.6.<br><br>The following Python packages were used for data visualization:<br>matplotlib 3.6.3<br>mpl_scatter_density 0.8<br>seaborn 0.13.2<br>shapely 2.0.6<br>scikit-image 0.21.0<br>scikit-learn 1.3.2<br>KDEpy 1.1.9<br>napari 0.4.18<br>napari-animation 0.0.7<br><br>Packages for cell segmentation:

stardist 0.9.1
cellpose 3.0.7

For manuscripts utilizing custom algorithms or software that are central to the research but not yet described in published literature, software must be made available to editors and reviewers. We strongly encourage code deposition in a community repository (e.g. GitHub). See the Nature Portfolio guidelines for submitting code & software for further information.

Data

Policy information about availability of data

All manuscripts must include a data availability statement. This statement should provide the following information, where applicable:

- Accession codes, unique identifiers, or web links for publicly available datasets
- A description of any restrictions on data availability
- For clinical datasets or third party data, please ensure that the statement adheres to our policy

The representative imaging dataset shown in Fig. 1 can be accessed via the Zenodo repository (https://zenodo.org/records/15599322). Due to the large file sizes, other imaging dataset will be made available upon request from the corresponding authors. Source data are provided with this paper.

Research involving human participants, their data, or biological material

Policy information about studies with human participants or human data. See also policy information about sex, gender (identity/presentation), and sexual orientation and race, ethnicity and racism.

Reporting on sex and gender	N/A
Reporting on race, ethnicity, or other socially relevant groupings	N/A
Population characteristics	N/A
Recruitment	N/A
Ethics oversight	N/A

Note that full information on the approval of the study protocol must also be provided in the manuscript.

Field-specific reporting

Please select the one below that is the best fit for your research. If you are not sure, read the appropriate sections before making your selection.

☒ Life sciences ☐ Behavioural & social sciences ☐ Ecological, evolutionary & environmental sciences

For a reference copy of the document with all sections, see nature.com/documents/nr-reporting-summary-flat.pdf

Life sciences study design

All studies must disclose on these points even when the disclosure is negative.

Sample size	No statistical methods were used to determine sample size. The number of mice per group within each independent experiment was determined based on similar experiments in the field, and on the practicality of conducting the animal study taking into account the total number of test conditions involved. In imaging experiments, 2-3 mice per group/condition were used. In flow cytometry experiments, 3-6 mice per group/condition were used. The exact numbers and statistical tests detailed in the figure legends and methods.
Data exclusions	No data were excluded from analysis.
Replication	Replicate experiments were performed with the number of independent experiments (minimum of 2) as detailed in the figure legends and methods sections.
Randomization	Age-matched littermate mice were used to control for litter, cage, and age effects. Mice were randomly assigned for experimental groups (but no specific randomization protocol was used).
Blinding	Investigators were not blinded to group allocation during data collection and analysis, as most of the readouts are quantitative and not subjective. Tumor injection, treatment and measurement were performed by one person and not blinded.

Reporting for specific materials, systems and methods

We require information from authors about some types of materials, experimental systems and methods used in many studies. Here, indicate whether each material, system or method listed is relevant to your study. If you are not sure if a list item applies to your research, read the appropriate section before selecting a response.

Materials & experimental systems

n/a	Involved in the study
☐	☒ Antibodies
☐	☒ Eukaryotic cell lines
☒	☐ Palaeontology and archaeology
☐	☒ Animals and other organisms
☒	☐ Clinical data
☒	☐ Dual use research of concern
☒	☐ Plants

Methods

n/a	Involved in the study
☒	☐ ChIP-seq
☐	☒ Flow cytometry
☒	☐ MRI-based neuroimaging

Antibodies

Antibodies used

All antibodies used including their conjugated fluorophores, clones, vendors, catalog numbers and dilutions used are listed in Supplementary Table 1.

Validation

All antibodies are validated per the data sheets available on the manufacturers' websites. For imaging experiments, validation was performed by assessing the immunostaining and their expected localization on the cells (membrane, cytoplasmic, nuclear) and the cell types as identified by other markers, on published immunohistochemistry data, and whenever possible, compared their expression level with the data obtained using flow cytometry (also presented in the extended data figures).

The following antibodies (clone, target species, application) were used and validation was performed as stated:

Rat monoclonal anti-PD-1 AF647 (RMP1-30, mouse, imaging), validation data from manufacturer's website: https://www.biolegend.com/en-us/products/alexa-fluor-647-anti-mouse-cd279-pd-1-antibody-12480

Rat monoclonal anti-PD-1 purified (RMP1-30, mouse, imaging), validation data from manufacturer's website: https://www.biolegend.com/en-us/products/purified-anti-mouse-cd279-pd-1-antibody-455

Rat monoclonal anti-CD45.1 AF594 (A20, mouse, imaging), validation data from manufacturer's website: https://www.biolegend.com/en-us/products/alexa-fluor-594-anti-mouse-cd45-1-antibody-13424

Rat monoclonal anti-CD45.2 AF594 (104, mouse, imaging), validation data from manufacturer's website: https://www.biolegend.com/en-us/products/alexa-fluor-594-anti-mouse-cd45-2-antibody-13446

Rat monoclonal anti-CD8b AF488 (YTS156.7.7, mouse, imaging), validation data from manufacturer's website: https://www.biolegend.com/en-us/products/alexa-fluor-488-anti-mouse-cd8b-ly-3-antibody-17365

Mouse monoclonal anti-Ly-108 AF647 (13G3, mouse, imaging), validation data from manufacturer's website: https://www.bdbiosciences.com/en-us/products/reagents/flow-cytometry-reagents/research-reagents/single-color-antibodies-ruo/alexa-fluor-647-mouse-anti-mouse-ly-108.561547

Mouse monoclonal anti-Ly-108 RY586 (13G3, mouse, imaging), validation data from manufacturer's website: https://www.bdbiosciences.com/en-us/products/reagents/flow-cytometry-reagents/research-reagents/single-color-antibodies-ruo/ry586-mouse-anti-mouse-ly-108.753676

Rat monoclonal anti-Ki-67 eFluor450 (SolA15, mouse, imaging), validation data from manufacturer's website: https://www.thermofisher.com/antibody/product/Ki-67-Antibody-clone-SolA15-Monoclonal/48-5698-82

Rabbit monoclonal anti-TCF-1 AF555 (C63D9, mouse, imaging), validation data from manufacturer's website: https://www.cellsignal.com/products/antibody-conjugates/tcf1-tcf7-c63d9-rabbit-mab-alexa-fluor-555-conjugate/17404

Rabbit monoclonal anti-TCF-1 AF594 (C63D9, mouse, imaging), validation data from manufacturer's website: https://www.cellsignal.com/products/antibody-conjugates/tcf1-tcf7-c63d9-rabbit-mab-alexa-fluor-594-conjugate/35972

Rabbit monoclonal anti-BATF AF647 (D7C5, mouse, imaging), validation data from manufacturer's website: https://www.cellsignal.com/products/antibody-conjugates/batf-d7c5-rabbit-mab-alexa-fluor-647-conjugate/47914

Rabbit monoclonal anti-BATF AF555 (D7C5, mouse, imaging), validation data from manufacturer's website: https://www.cellsignal.com/products/primary-antibodies/batf-d7c5-rabbit-mab/8638. Additional validation by comparing with AF647-conjugated anti-BATF antibody.

Rabbit monoclonal anti-NFAT1 AF647 (D43B1, mouse, imaging), validation data from manufacturer's website: https://www.cellsignal.com/products/antibody-conjugates/nfat1-d43b1-xp-rabbit-mab-alexa-fluor-647-conjugate/14201

Rabbit monoclonal anti-cleaved caspase-3 (Asp175) AF555 (D3E9, mouse, imaging), validation data from manufacturer's website: https://www.cellsignal.com/products/antibody-conjugates/cleaved-caspase-3-asp175-d3e9-rabbit-mab-alexa-fluor-555-conjugate/9604

Goat polyclonal anti-PD-L1 (polyclonal, mouse, imaging), validation data from manufacturer's website: https://www.rndsystems.com/products/mouse-pd-l1-b7-h1-antibody_af1019

Rabbit polyclonal anti-XCR1 (polyclonal, mouse, imaging), validation data from manufacturer's website: https://www.lsbio.com/antibodies/xcr1-antibody-aa244-322-wb-western-ls-c763561/789657. Additional validation performed on lymph node tissues from XCR1-venus reporter mice (heterozygous XCR1-venus knock-in for presence of XCR1 protein, and homozygous knock-in for absence of XCR1).

Rat monoclonal anti-CD3 BV711 (17A2, mouse, flow cytometry), validation data from manufacturer's website: https://www.biolegend.com/en-us/products/brilliant-violet-711-anti-mouse-cd3-antibody-10022

Armenian Hamster monoclonal anti-CD27 BV650 (LG.3A10, mouse, flow cytometry), validation data from manufacturer's website: https://www.biolegend.com/en-us/products/brilliant-violet-650-anti-mouse-rat-human-cd27-antibody-14413

Rat monoclonal anti-PD-1 BV785 (29F.1A12, mouse, flow cytometry), validation data from manufacturer's website: https://www.biolegend.com/en-us/products/brilliant-violet-785-anti-mouse-cd279-pd-1-antibody-9874

Rat monoclonal anti-CD200 PE-Cy7 (OX-90, mouse, flow cytometry), validation data from manufacturer's website: https://www.biolegend.com/en-us/products/pecyanine7-anti-mouse-cd200-ox2-antibody-19211

Rat monoclonal anti-CD44 AF700 (IM7, mouse, flow cytometry), validation data from manufacturer's website: https://www.biolegend.com/en-us/products/alexa-fluor-700-anti-mouse-human-cd44-antibody-3406

Rat monoclonal anti-PD-L1 BV421 (10F.9G2, mouse, flow cytometry), validation data from manufacturer's website: https://www.biolegend.com/en-us/products/brilliant-violet-421-anti-mouse-cd274-b7-h1-pd-l1-antibody-7250

Rat monoclonal anti-PD-L2 PE-Dazzle 594 (TY25, mouse, flow cytometry), validation data from manufacturer's website: https://www.biolegend.com/en-us/products/pedazzle594-anti-mouse-cd273-antibody-15630

Rat monoclonal anti-Vα2 PE-Cy7 (B20.1, mouse, flow cytometry), validation data from manufacturer's website: https://www.biolegend.com/en-us/products/pe-cyanine7-anti-mouse-tcr-va2-antibody-15019

Rat monoclonal anti-CD45.1 AF488 (A20, mouse, flow cytometry), validation data from manufacturer's website: https://www.biolegend.com/en-us/products/alexa-fluor-488-anti-mouse-cd45-1-antibody-3103

Rat monoclonal anti-CD45.1 AF700 (A20, mouse, flow cytometry), validation data from manufacturer's website: https://www.biolegend.com/en-us/products/alexa-fluor-700-anti-mouse-cd45-1-antibody-3392

Rat monoclonal anti-CD45.2 AF488 (104, mouse, flow cytometry), validation data from manufacturer's website: https://www.biolegend.com/en-us/products/alexa-fluor-488-anti-mouse-cd45-2-antibody-3106

Rat monoclonal anti-CD45.2 AF647 (104, mouse, flow cytometry), validation data from manufacturer's website: https://www.biolegend.com/en-us/products/alexa-fluor-647-anti-mouse-cd45-2-antibody-3107

Mouse monoclonal anti-Bcl-2 AF488 (BCL/10C4, mouse, flow cytometry), validation data from manufacturer's website: https://www.biolegend.com/en-us/products/alexa-fluor-488-anti-bcl-2-antibody-6346

Mouse monoclonal anti-T-bet PE-Cy7 (4B10, mouse, flow cytometry), validation data from manufacturer's website: https://www.biolegend.com/en-us/products/pe-cyanine7-anti-t-bet-antibody-8328

Rat monoclonal anti-MHC-II (I-A/I-E) AF700 (M5/114.15.2, mouse, flow cytometry), validation data from manufacturer's website: https://www.biolegend.com/en-us/products/alexa-fluor-700-anti-mouse-i-a-i-e-antibody-3413

Armenian Hamster monoclonal anti-CD11c PE-Cy7 (N418, mouse, flow cytometry), validation data from manufacturer's website: https://www.biolegend.com/en-us/products/pe-cyanine7-anti-mouse-cd11c-antibody-3086

Rat monoclonal anti-SIRPα AF488 (P84, mouse, flow cytometry), validation data from manufacturer's website: https://www.biolegend.com/en-us/products/alexa-fluor-488-anti-mouse-cd172a-sirpalpha-antibody-14089

Mouse monoclonal anti-XCR1 PE (ZET, mouse, flow cytometry), validation data from manufacturer's website: https://www.biolegend.com/en-us/products/pe-anti-mouse-rat-xcr1-antibody-10217

Mouse monoclonal anti-XCR1 AF647 (ZET, mouse, flow cytometry), validation data from manufacturer's website: https://www.biolegend.com/en-us/products/alexa-fluor-647-anti-mouse-rat-xcr1-antibody-10402

Armenian Hamster monoclonal anti-CD80 PerCP-Cy5.5 (16-10A1, mouse, flow cytometry), validation data from manufacturer's website: https://www.biolegend.com/en-us/products/percp-cyanine5-5-anti-mouse-cd80-antibody-4275

Mouse monoclonal anti-CX3CR1 BV421 (SA011F11, mouse, flow cytometry), validation data from manufacturer's website: https://www.biolegend.com/en-us/products/brilliant-violet-421-anti-mouse-cx3cr1-antibody-11852

Mouse monoclonal anti-CX3CR1 BV605 (SA011F11, mouse, flow cytometry), validation data from manufacturer's website: https://www.biolegend.com/en-us/products/brilliant-violet-605-anti-mouse-cx3cr1-antibody-12110

Rat monoclonal anti-CD39 PE/Dazzle™ 594 (Duha59, mouse, flow cytometry), validation data from manufacturer's website: https://

www.biolegend.com/en-us/products/pe-dazzle-594-anti-mouse-cd39-antibody-16385

Mouse monoclonal anti-H-2Kb bound to SIINFEKL APC (25-D1.16, mouse, flow cytometry), validation data from manufacturer's website: https://www.biolegend.com/en-us/products/apc-anti-mouse-h-2kb-bound-to-siinfekl-antibody-7882

Rat monoclonal anti-CD8α BUV395 (53-6.7, mouse, flow cytometry), validation data from manufacturer's website: https://www.bdbiosciences.com/en-us/products/reagents/flow-cytometry-reagents/research-reagents/single-color-antibodies-ruo/buv395-rat-anti-mouse-cd8a.563786

Mouse monoclonal anti-Ly-108 BUV737 (13G3, mouse, flow cytometry), validation data from manufacturer's website: https://www.bdbiosciences.com/en-us/products/reagents/flow-cytometry-reagents/research-reagents/single-color-antibodies-ruo/buv737-mouse-anti-mouse-ly-108.741893

Rat monoclonal anti-CD24 BUV737 (M1/69, mouse, flow cytometry), validation data from manufacturer's website: https://www.bdbiosciences.com/en-us/products/reagents/flow-cytometry-reagents/research-reagents/single-color-antibodies-ruo/buv737-rat-anti-mouse-cd24.612832

Rat monoclonal anti-CD4 BV786 (RM4-4, mouse, flow cytometry), validation data from manufacturer's website: https://www.bdbiosciences.com/en-us/products/reagents/flow-cytometry-reagents/research-reagents/single-color-antibodies-ruo/bv786-rat-anti-mouse-cd4.740844

Rat monoclonal anti-Ki-67 AF488 (SolA15, mouse, flow cytometry), validation data from manufacturer's website: https://www.thermofisher.com/antibody/product/Ki-67-Antibody-clone-SolA15-Monoclonal/53-5698-82

Rabbit monoclonal anti-TCF-1 AF647 (C63D9, mouse, flow cytometry), validation data from manufacturer's website: https://www.cellsignal.com/products/antibody-conjugates/tcf1-tcf7-c63d9-rabbit-mab-alexa-fluor-647-conjugate/6709

Rabbit monoclonal anti-BATF AF647 (D7C5, mouse, flow cytometry), validation data from manufacturer's website: https://www.cellsignal.com/products/antibody-conjugates/batf-d7c5-rabbit-mab-alexa-fluor-647-conjugate/47914

Rabbit monoclonal anti-BATF AF555 (D7C5, mouse, flow cytometry), validation data from manufacturer's website: https://www.cellsignal.com/products/primary-antibodies/batf-d7c5-rabbit-mab/8638. Additional validation by comparing with AF647-conjugated anti-BATF antibody.

Rabbit monoclonal anti-cleaved caspase-3 (Asp175) AF488 (D3E9, mouse, flow cytometry), validation data from manufacturer's website: https://www.cellsignal.com/products/antibody-conjugates/cleaved-caspase-3-asp175-d3e9-rabbit-mab-alexa-fluor-488-conjugate/9603

Rabbit monoclonal anti-Ubiquityl-Histone H2A (Lys119) AF488 (D27C4, mouse, flow cytometry), validation data from manufacturer's website: https://www.cellsignal.com/products/antibody-conjugates/ubiquityl-histone-h2a-lys119-d27c4-xp-rabbit-mab-alexa-fluor-488-conjugate/26498. Antibody chosen based on Kang et al., 2024: 10.1126/science.adl4492.

Eukaryotic cell lines

Policy information about cell lines and Sex and Gender in Research

Cell line source(s)	KP1233 (from T. Jacks lab) and MC38 (from M. Meier-Schellersheim lab) were mouse derived tumor cell lines. 293T adherent (Takara #632180, gift from T. Jacks lab) cells were used for lentiviral production.
Authentication	No authentication was performed beyond that provided by the laboratories who gifted the cell lines, or by the vendors.
Mycoplasma contamination	293T lines were tested negative for mycoplasma. Tumor cell lines were not tested for mycoplasma.
Commonly misidentified lines (See ICLAC register)	No commonly misidentified cell lines were used.

Animals and other research organisms

Policy information about studies involving animals; ARRIVE guidelines recommended for reporting animal research, and Sex and Gender in Research

Laboratory animals

Species, strains and age information of mice used in this study are detailed in the methods section. Mice within individual experiments were age and sex matched.

All mice were housed in 12-hour light-dark cycle with ad libitum access to food and water.
CD45.2 (C57BL/6J) and B6.GFP (C57BL/6-Tg(UBC-GFP)30Scha/J) mice were purchased from the Jackson Laboratory (strain numbers 000664 and 004354 respectively); CD45.1 (B6.SJL Ptprca), OT-I.CD45.1 (B6(Ly5.1)-[Tg]TCR OT-I-[KO]RAG1), OT-I.CD45.2 (C57BL/6NAi-[Tg]TCR OT-1-[KO]RAG1) were obtained from the NIAID-Taconic exchange program (strain numbers 8478, 300 and 175, respectively). XCR1-DTR (B6.Cg-Xcr1tm2(HBEGF/Venus)Ksho) and XCR1-venus (B6.Cg-Xcr1tm1Ksho) transgenic mice15 were kind gifts from Tsuneyasu Kaisho (RIKEN-Yokohama and Osaka University). OT-I.GFP mice were cross-bred from OT-I.CD45.2 and B6.GFP and maintained as a homozygous strain in our laboratory. The majority of mice employed were female and aged 6-16 weeks at the beginning of experiments, with a small number of experiments performed in male mice. All mice were bred and maintained under

	specific pathogen-free conditions at an American Association for the Accreditation of Laboratory Animal Care (AAALAC)-accredited animal facility within NIAID and were used under study protocol LISB-4E approved by NIAID Animal Care and Use Committee (NIH).
Wild animals	No wild animals were used in this study.
Reporting on sex	The majority of mice used for tumor studies were female mice, with a small number of experiments performed using male mice. No significant difference was observed between sex.
Field-collected samples	No samples collected from the filed were used in this study.
Ethics oversight	All protocols were approved by NIAID Animal Care and Use Committee (NIH) at an American Association for the Accreditation of Laboratory Animal Care (AAALAC)-accredited animal facility within NIAID .

Note that full information on the approval of the study protocol must also be provided in the manuscript.

Plants

Seed stocks	*Report on the source of all seed stocks or other plant material used. If applicable, state the seed stock centre and catalogue number. If plant specimens were collected from the field, describe the collection location, date and sampling procedures.*
Novel plant genotypes	*Describe the methods by which all novel plant genotypes were produced. This includes those generated by transgenic approaches, gene editing, chemical/radiation-based mutagenesis and hybridization. For transgenic lines, describe the transformation method, the number of independent lines analyzed and the generation upon which experiments were performed. For gene-edited lines, describe the editor used, the endogenous sequence targeted for editing, the targeting guide RNA sequence (if applicable) and how the editor was applied.*
Authentication	*Describe any authentication procedures for each seed stock used or novel genotype generated. Describe any experiments used to assess the effect of a mutation and, where applicable, how potential secondary effects (e.g. second site T-DNA insertions, mosiacism, off-target gene editing) were examined.*

Flow Cytometry

Plots

Confirm that:

☒ The axis labels state the marker and fluorochrome used (e.g. CD4-FITC).

☒ The axis scales are clearly visible. Include numbers along axes only for bottom left plot of group (a 'group' is an analysis of identical markers).

☒ All plots are contour plots with outliers or pseudocolor plots.

☒ A numerical value for number of cells or percentage (with statistics) is provided.

Methodology

Sample preparation	Lymphocytes were isolated from lymph nodes and spleens and made into single-cell suspensions using a syringe plunger and 100µm or 70µm cell strainers (MACS SmartStrainer, Miltenyi Biotec). ~3x106 cells were used in subsequent staining steps for flow cytometry analysis. Dendritic cell isolation was performed as described previously. Briefly, lymphoid tissues were sliced into small fragments using a scalpel blade and incubated in a digestion mix of collagenase type III (Worthington, 1mg/ml) and DNase I (20ug/ml) and vigorously mixed for 25 min at room temperature, followed by addition of 0.1M EDTA solution at 1/10 digestion volume for 5 min to dissociate lymphocytes from dendritic cells. Tissue debris were filtered out by passing the cell suspension through a 70µm nylon mesh.<br><br>T cells from skin tumor samples were isolated as described previously. Briefly, a 1cm2x1cm2 tumor-containing skin patch was harvested into collagenase type III (Worthington, 3mg/ml), finely chopped with scissors and incubated at 37°C for 90 min before pressing through 70µm cell strainers. For spleen and tumor samples, cells were also treated with red blood cell lysis buffer prior to staining. Cell counts in LN and spleen were determined using an automated Cellometer T4 cell counter (Nexcelom Bioscience).<br><br>For detection of polyclonal OVA-specific CD8+ T cells, cells were first incubated with PE-conjugated H-2Kb-SIINFEKL tetramer (1:100, NIH Tetramer Core) for 20 min at 37°C, washed, followed by cell surface marker staining for 25 min at 4°C. Mouse BD Fc Block (1:200; BD Biosciences) was also included during cell surface marker staining step. A fixable LIVE/DEAD near-infrared staining dye was used for determining cell viability. For detection of intracellular proteins, stained cells were further treated with fixative and stained for antibodies against intracellular proteins using Foxp3/transcription factor staining buffer kit per the manufacturer's protocol (eBioscience). For tumor samples, CountBright Plus Absolute beads (Thermofisher) were added prior to sample acquisition. Samples were acquired using a BD Fortessa (BD Biosciences) and analyzed using Flowjo 10 (Treestar).
Instrument	BD Fortessa was used for flow cytometry data acquisition.
Software	FlowJo v10

Cell population abundance	Frequencies of cell populations are indicated on flow cytometry plots and their absolute count quantified.
Gating strategy	Antigen-specific lymphocytes were gated based on LIVE/DEAD stain, CD3 and CD8a, and then gated using the appropriate tetramers. PD-1+ gate was used to gate on activated lymphocytes and filter out rare, contaminating population. Gating strategy for further subsetting are shown in Figures and Extended Data Figures.

☒ Tick this box to confirm that a figure exemplifying the gating strategy is provided in the Supplementary Information.
