# Supplementary figures and images for: Inhibitory PD-1 axis maintains high-avidity stem-like CD8+ T cells
Source: Nature. 2025 Nov 26;649(8095):194–204. doi: 10.1038/s41586-025-09440-x (PMC12727512; doi:10.1038/s41586-025-09440-x)

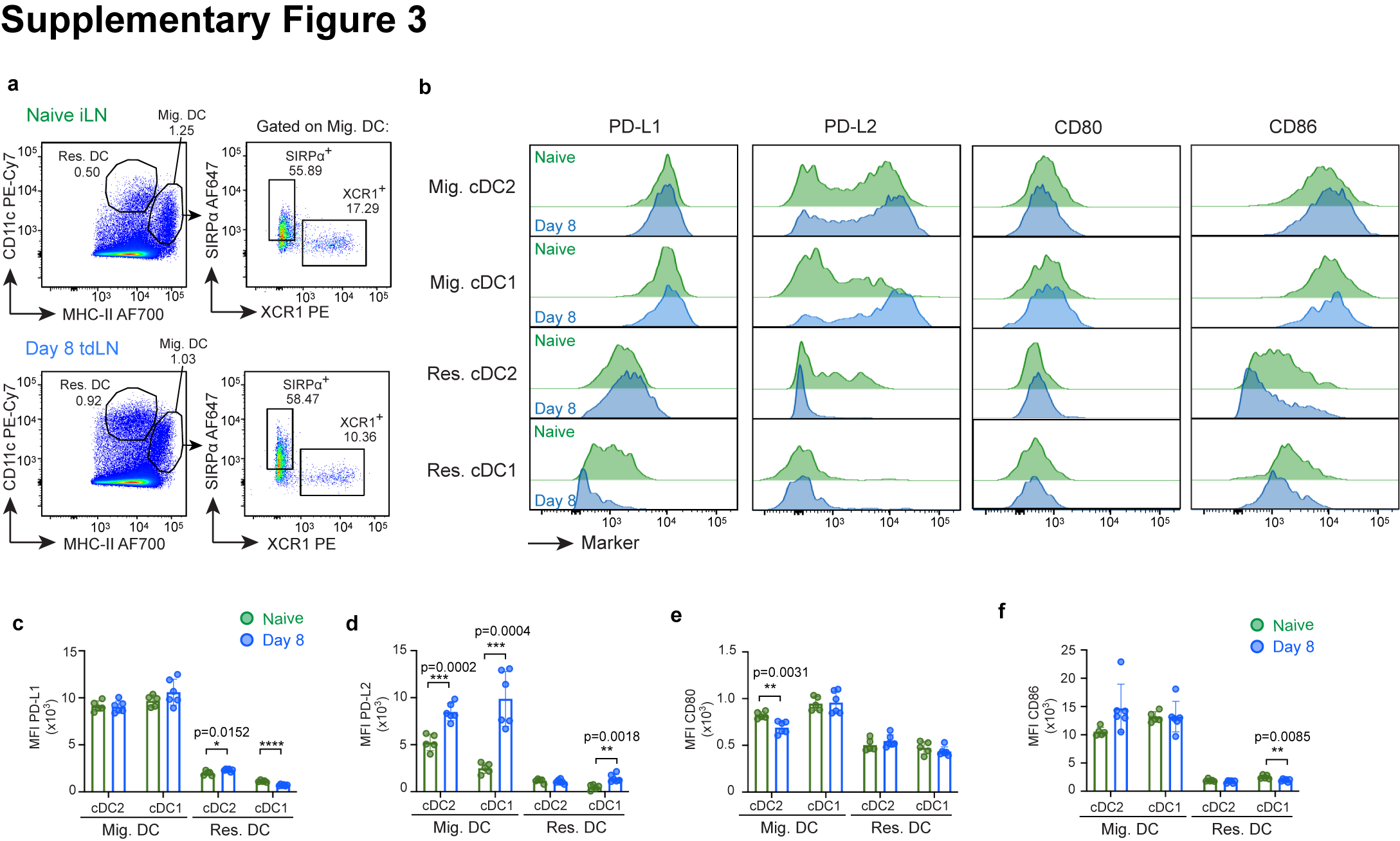

Supplement: Supplementary file 3 — Supplementary Fig. 1: the flow cytometric gating strategy of OVA-antigen specific CD8+ T cells. Gating strategy for OVA–tetramer+CD8+ T cells in tdLN. Supplementary Fig. 2: characterization of cDC1 cell depletion in XCR1-DTR mice. a, Experimental scheme of diphtheria toxin (DT) administration in mice transferred with OT-I cells and received intradermal OVA/poly(I:C) immunization. b, Full 300-µm-thickness view of day 6 immunized inguinal draining lymph node stained with polyclonal anti-XCR1 antibodies (green) to assess the depletion efficiency. c, Magnified images of OT-I cell clustering around one of the few remaining cDC1 cells, showing PD-1 (left) and TCF-1 (right) expression masked on OT-I. The dotted yellow box denotes a magnified region shown in the bottom row. Supplementary Fig. 3: co-stimulatory and co-inhibitory ligand expression by cDC subsets in tdLN. a, Flow cytometry gating strategy of migratory versus lymph node-resident dendritic cell subsets isolated from naive (top) or day 8 tdLN (bottom) based on CD11c and MHC-II expression (left), and further into SIRPα+ cDC2 cells and XCR1+ cDC1 cells (right). Histogram panels showing relative expression of PD-L1, PD-L2, CD80 and CD86 markers on each cDC subset are shown in b and the quantification of the MFI for each marker in c–f. Data from two independent experiments (n = 5 (naive), 6 (day 8 tdLN)). Data are mean ± s.d. Statistical analysis was performed using unpaired two-tailed t-tests; *P < 0.05, **P < 0.01, ***P < 0.001. [file 41586_2025_9440_MOESM3_ESM.zip › Supplementary Figures/Supplementary_Fig_3.tif]

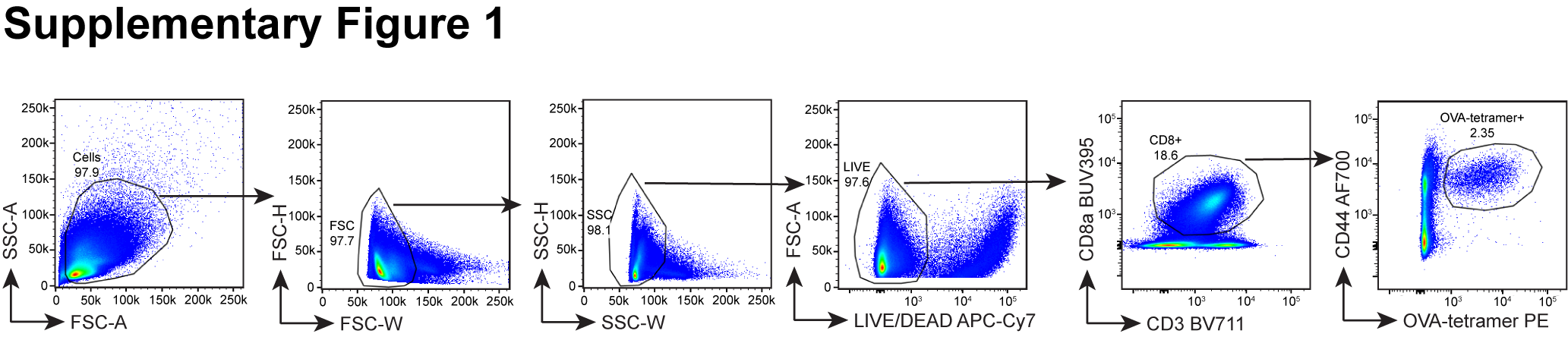

Supplement: Supplementary file 3 — Supplementary Fig. 1: the flow cytometric gating strategy of OVA-antigen specific CD8+ T cells. Gating strategy for OVA–tetramer+CD8+ T cells in tdLN. Supplementary Fig. 2: characterization of cDC1 cell depletion in XCR1-DTR mice. a, Experimental scheme of diphtheria toxin (DT) administration in mice transferred with OT-I cells and received intradermal OVA/poly(I:C) immunization. b, Full 300-µm-thickness view of day 6 immunized inguinal draining lymph node stained with polyclonal anti-XCR1 antibodies (green) to assess the depletion efficiency. c, Magnified images of OT-I cell clustering around one of the few remaining cDC1 cells, showing PD-1 (left) and TCF-1 (right) expression masked on OT-I. The dotted yellow box denotes a magnified region shown in the bottom row. Supplementary Fig. 3: co-stimulatory and co-inhibitory ligand expression by cDC subsets in tdLN. a, Flow cytometry gating strategy of migratory versus lymph node-resident dendritic cell subsets isolated from naive (top) or day 8 tdLN (bottom) based on CD11c and MHC-II expression (left), and further into SIRPα+ cDC2 cells and XCR1+ cDC1 cells (right). Histogram panels showing relative expression of PD-L1, PD-L2, CD80 and CD86 markers on each cDC subset are shown in b and the quantification of the MFI for each marker in c–f. Data from two independent experiments (n = 5 (naive), 6 (day 8 tdLN)). Data are mean ± s.d. Statistical analysis was performed using unpaired two-tailed t-tests; *P < 0.05, **P < 0.01, ***P < 0.001. [file 41586_2025_9440_MOESM3_ESM.zip › Supplementary Figures/Supplementary_Fig_1.tif]

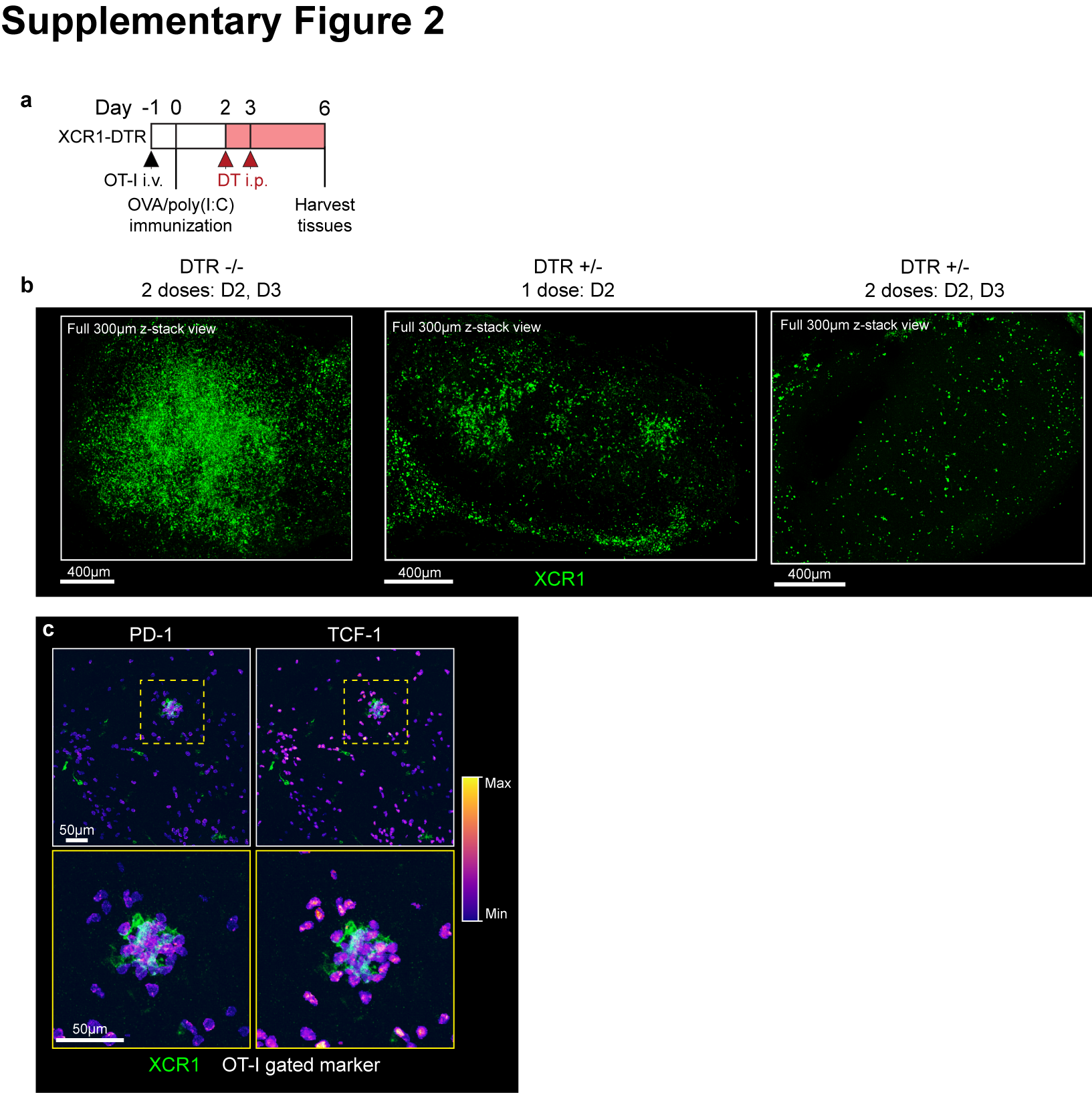

Supplement: Supplementary file 3 — Supplementary Fig. 1: the flow cytometric gating strategy of OVA-antigen specific CD8+ T cells. Gating strategy for OVA–tetramer+CD8+ T cells in tdLN. Supplementary Fig. 2: characterization of cDC1 cell depletion in XCR1-DTR mice. a, Experimental scheme of diphtheria toxin (DT) administration in mice transferred with OT-I cells and received intradermal OVA/poly(I:C) immunization. b, Full 300-µm-thickness view of day 6 immunized inguinal draining lymph node stained with polyclonal anti-XCR1 antibodies (green) to assess the depletion efficiency. c, Magnified images of OT-I cell clustering around one of the few remaining cDC1 cells, showing PD-1 (left) and TCF-1 (right) expression masked on OT-I. The dotted yellow box denotes a magnified region shown in the bottom row. Supplementary Fig. 3: co-stimulatory and co-inhibitory ligand expression by cDC subsets in tdLN. a, Flow cytometry gating strategy of migratory versus lymph node-resident dendritic cell subsets isolated from naive (top) or day 8 tdLN (bottom) based on CD11c and MHC-II expression (left), and further into SIRPα+ cDC2 cells and XCR1+ cDC1 cells (right). Histogram panels showing relative expression of PD-L1, PD-L2, CD80 and CD86 markers on each cDC subset are shown in b and the quantification of the MFI for each marker in c–f. Data from two independent experiments (n = 5 (naive), 6 (day 8 tdLN)). Data are mean ± s.d. Statistical analysis was performed using unpaired two-tailed t-tests; *P < 0.05, **P < 0.01, ***P < 0.001. [file 41586_2025_9440_MOESM3_ESM.zip › Supplementary Figures/Supplementary_Fig_2.tif]
